# Reprogrammable Metasurface Controlled by 2D Thermal Fields

**DOI:** 10.3390/mi13112023

**Published:** 2022-11-19

**Authors:** Ming Zhang, Fuju Ye, Hongrui Tan, Sisi Luo, Haoyang Cui, Lei Chen

**Affiliations:** College of Electronics and Information Engineering, Shanghai University of Electric Power, No. 2588 Changyang Road, Yangpu District, Shanghai 200090, China

**Keywords:** thermal-sensing, reprogrammable, metasurface

## Abstract

The combination of thermal field sensing and microwave operation is an innovative topic in metamaterials. Although there exists research on modulating electromagnetic waves by controlling each column of the metasurface elements for programmable metasurfaces, the regulation is not flexible. In view of this, this paper proposes a metasurface based on distributed thermal sensing that can be independently modulated by each element. In this paper, the metasurface adopts a 1-bit coding metasurface, which is combined with PIN diodes to modulate the phase response. The voltage control circuit feeds back the change in the thermistors to the switching state of the PIN diode. Each metasurface unit contains thermistors, which are used to sense thermal stimulation and can be independently modulated. The metasurface composed of these elements can feel the field generated via heat energy. We can control electromagnetic waves by controlling this field. In order to prove the feasibility of this scheme, a metasurface sample of 8 × 8 elements was designed. Three patterns were used for the design, fabrication, and measurement of the samples. Meanwhile, printed circuit board (PCB) technology was applied. The results show that the simulated results are highly consistent with the experimental results, which verifies that this scheme is practicable.

## 1. Introduction

Metamaterials are artificially designed composite materials that possess an excellent ability to modulate electromagnetic waves [1]. Since they have excellent properties that do not exist in nature, such as negative refraction and a zero refractive index, metamaterials have attracted extensive research and a plethora of applications, including optical lenses [2], cloaks [3,4], and imaging systems [5,6,7]. Metasurfaces are 2D versions of metamaterials, which are ultra-thin planes of subwavelength metal or dielectric cells with specific electromagnetic wave modification capabilities, such as phase and amplitude modulation. Compared to metamaterials, metasurfaces have the advantages of low profile, low loss, and easy processing.

The notion of the reprogrammable metasurface is proposed to meet the needs of many practical applications [8,9,10,11,12,13]. For example, it is applied to high-sensitivity optical sensors, miniaturized image sensors, consumer electronics, and sophisticated medical equipment that require strict packaging [14,15,16,17]. The combination of reprogrammable metasurfaces with adjustable components [18,19], such as positive-intrinsic-negative (PIN) diodes and varactors [20,21], increases the degree of freedom of phase control over the metasurface to achieve dynamically modulating electromagnetic waves [22,23,24,25,26,27,28,29]. By integrating sensing components onto reprogrammable metasurfaces [30,31], reprogrammable sensing metasurfaces connect distinct physical and electromagnetic fields [32], broadening the idea of regulating multiphysical fields like light control [33,34,35] and thermal regulation [36,37,38]. However, most previously proposed metasurface research on thermal control has focused on engineering the manipulation of absorption qualities or thermal emissions or the phase modulation in columns, although the accurate phase regulation within units has not been presented or researched.

In this paper, we propose a reprogrammable metasurface controlled by 2D thermal fields; the minimum regulated unit is the element. We first connect each metamaterial unit with the microcircuit to form a closed circuit, where the thermally-sensitive resistance senses the thermal stimulus, and the PIN diode outputs different voltage values. Secondly, the elements are arranged into 2D planes, and the microcircuits and the metasurface are integrated into the PCB to modulate the phase distribution of the metasurface. In order to verify our idea, we designed three different coding patterns for the simulation and fabrication of the metasurface for experimental testing. The experimental test results were highly consistent with the simulation results. We envisage this work as a new way of carrying out thermal sensing and microwave manipulation.

## 2. Principle and Results

**Figure 1:**
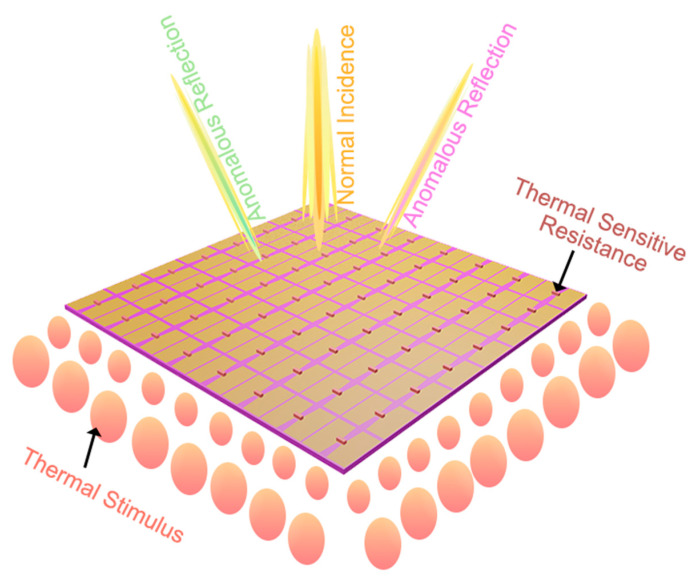
Schematic illustration of a temperature-sensitive metasurface. When a metasurface containing a thermistor experiences an external thermal stimulus, its reflection phase pattern is reprogrammed, resulting in anomalous reflection fields.

Figure 1 is a schematic diagram of the proposed thermal-sensing metasurface, which combines the 1-bit programmable metasurface embedded with the PIN diodes and thermistors. The metasurface element embedded with the PIN diodes is used to modulate the reflection phase response and can reflect various scattering fields under different thermal stimuli. Each unit cell of the metasurface can be regulated because each element contains a thermistor to sense the thermal stimulation. The metasurface, which is composed of such units, can sense the field generated by thermal energy. We control this field to regulate electromagnetic waves. At the same time, the control of the thermal field can be achieved by regulating the arbitrary elements of the metasurface. In this way, the thermal field and electromagnetic wave are combined to realize the deflection of the different beams on the metasurface. The thermistor can sense the variation of external temperature, and the resistance value change with the change of temperature. This change in electrical characteristics will be reflected in the voltage control module, thus affecting the bias voltage of the PIN diodes. At this time, the related elements will switch the reflected phase response, resulting in various abnormal reflections on the metasurface.

**Figure 2:**
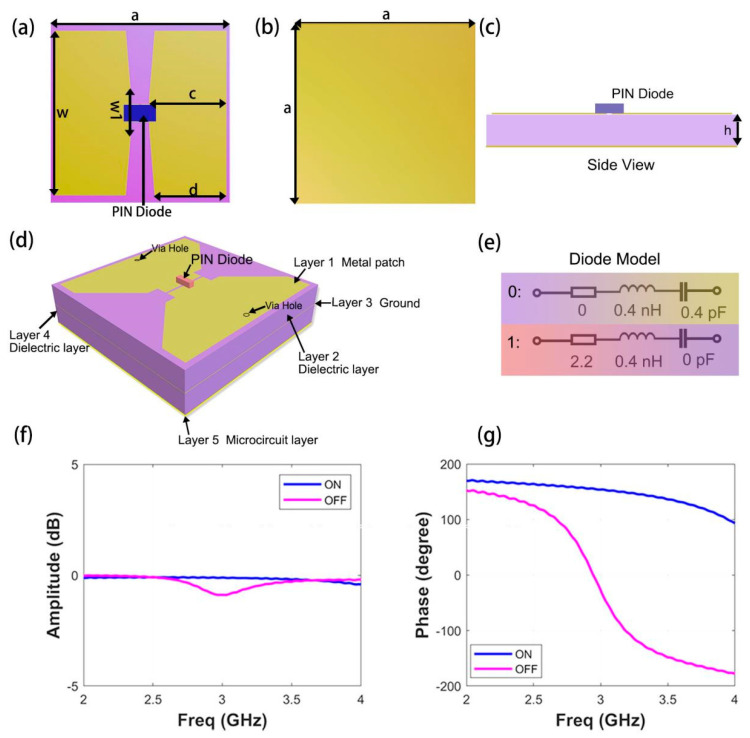
Illustration of the element used for wavefront manipulation and electromagnetic response, and the schematic diagram of metasurface structure. (**a**) Three-dimensional perspective view of the element; (**b**) back view of the element; (**c**) side view of the element; (**d**) schematic diagram of the five-layer structure of the fabricated sample; (**e**) equivalent circuit of PIN diode in OFF/ON state; (**f**) magnitude response of the element; (**g**) phase response of the element.

The metasurface element used is illustrated in Figure 2a–c. The top and bottom layers are metal layers, and the middle is a 3 mm dielectric substrate (F4B) with a dielectric constant of 3.0 and a loss tangent of 0.002. Two symmetrical metal patches bridged by a PIN diode (Skyworks SMP1320, skyworks solutions inc., Shenzhen, China.) are embedded at both ends of the top metal layer. The PIN diode is regarded as an RLC model during the simulation, and the switching time is 1 μs. There are “via” holes on the metal patches at both ends, which are used to provide a DC bias to control the on/off state of the diode. For more effective modulation, we use the numerical simulation software CST Microwave Studio (CST Microwave Studio, version 2020, accessed on 14 July 2022) to optimize the structure. The period of the metasurface element is *a* = 30 mm, and the geometric dimensions of the metal patches were selected as follows: *w* = 27.5 mm, *c* = 12.9 mm, and *d* = 12 mm. The Linear y-polarized normal incidence plane was used in the experiment. Meanwhile, the element with the diode was encoded as “0” in the OFF state and “1” in the ON state.

Figure 2f,g show the simulation results of the reflected EM response when different voltages were applied to the PIN diode. The numeric states “0” and “1” are marked in purple and blue, respectively. By changing the geometric parameters (*w*, *w1*, *c*, and *d*) of the metal patch, better electromagnetic characteristics were obtained on the premise that the phase difference is 180°. The simulated phase response shows that the phase difference (of 180°) is achieved at 3 GHz, and the correlation amplitude is −0.11 dB and −0.73 dB, respectively. The proposed metasurface structure is shown in Figure 2d, and the equivalent diagram of the PIN diode is shown in Figure 2e.

**Figure 3:**
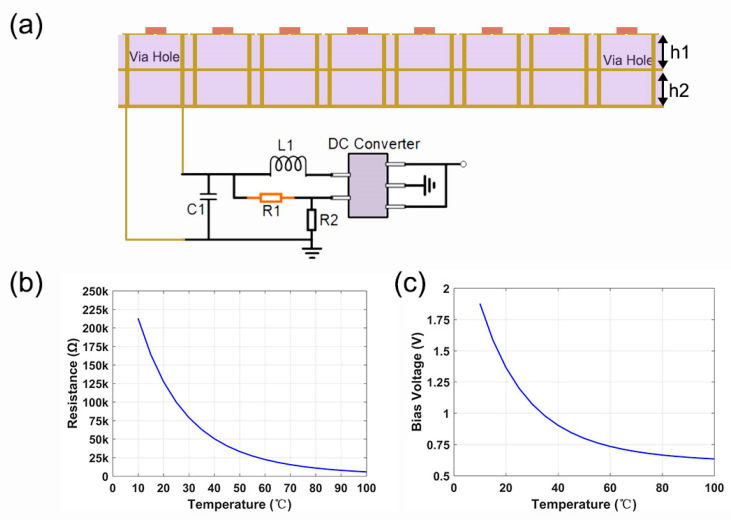
The design diagram of microcircuit, and a graphic of resistance value and bias voltage changing with temperature. (**a**) Specific voltage control module diagram; (**b**,**c**) show the schematic diagram of the change in thermistor and bias voltage with temperature.

To realizing effective regulation and modulation according to temperature changes, we have meticulously designed a manufactured sample with a five-layer structure. The top layer is a metal patch, the third layer is metal ground, the bottom layer is a microcircuit layer, and the remaining two layers are dielectric layers. The role of the “via” hole is to connect each layer as a bridge. The metal patches at both ends of the metasurface element output different voltage values as the input to the microcircuit. Each cell on the metasurface contains thermistors, which can sense thermal stimulation and achieve the function whereby every point can be modulated. Note that the diode was regarded as an RLC model in the simulation. The variation in thermal resistance will cause the corresponding change in the PIN diode voltage, which makes the PIN diodes switch between the on and off states, thus causing the working state of the metasurface to change, leading to the metasurface engender distinct phase response. As illustrated in Figure 3a, in the specific microcircuit, the feedback network of the DC converter (tps62240, Shenzhen Smach Technology Co., LTD, Shenzhen, China.) has two electric resistances which are used to alter the output voltage. The thermistor marked in red adopts a negative temperature coefficient (NTC) with a B value of 4250, and its resistance changes with temperature. As shown in Figure 3b,c, when the temperature rises from 25 °C to 100 °C, the resistance can be observed to drop from 100 KΩ to about 5 KΩ. If the temperature rises above 40 °C, the diode will turn off, and the metasurface will work in the “0” state at this time. Another fixed resistance value is 100 KΩ. The metasurface is encoded by the same number of coding elements, 8 × 8 (8 elements along the x-axis), with 64 diodes integrated. Each unit cell shares the same bias voltage and has the same phase response. 

**Figure 4:**
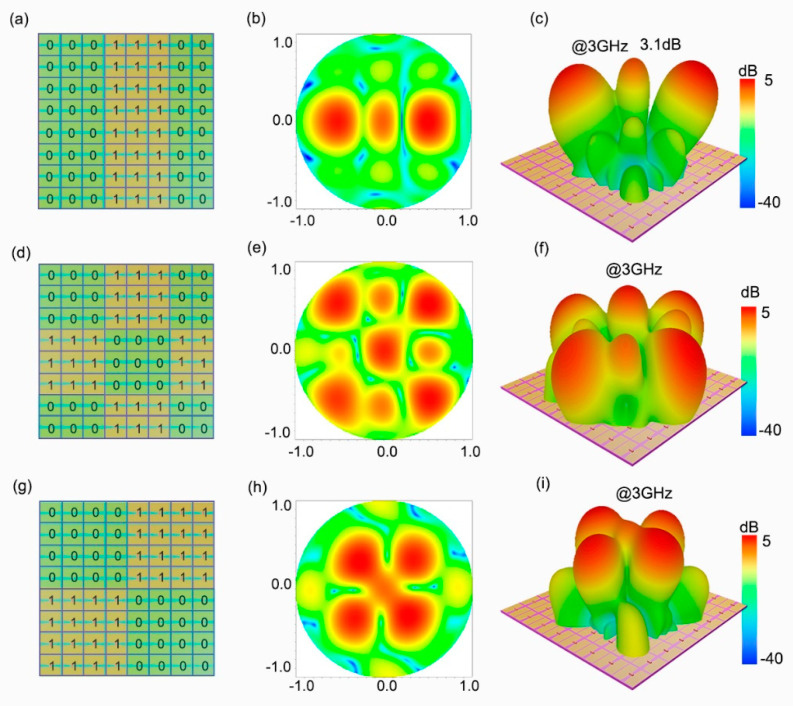
Three coding patterns of the metasurface and simulated far-field results at 3 GHz. (**a**) The coding sequence of pattern one is “00011100”; (**d**) pattern two has the horizontal coding sequence “00011100”, and (**g**) the horizontal coding sequence of pattern three is “00001111”; (**b**,**c**,**e**,**f**,**h**,**i**) are the simulated far-field results corresponding to the three patterns.

As shown in Figure 4a,d,g, we encode the metasurface into the following three patterns. In pattern one, the metasurface array coding sequence is “00011100”. The horizontal coding and vertical coding sequences in pattern two and pattern three are also the same. Pattern two is “00011100”, while pattern three is “00001111”. Under the plane wave incidence, diverse beam deflection phenomena will appear on the metasurface with various patterns. As indicated in Figure 4b,c, pattern one reflects the incident wave into a dual-beam with the deflection angle θ = ± 30° at 3 GHz, and the energy is mainly concentrated into the two side beams. In Figure 4e,f,h,i, patterns 2 and 3 reflect the incident wave into four beams at 3 GHz, respectively. By comparing the simulated scattering field results of the three patterns, it can be seen that most of the energy is concentrated into the side beam, and from pattern one to pattern three, the deflection angle becomes smaller and smaller.

**Figure 5:**
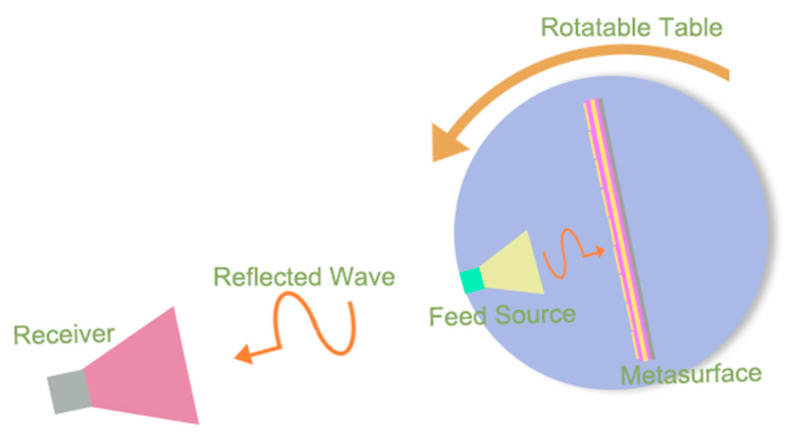
The experimental environment of the far-field test.

In this experimental demonstration, the far-field measurement of the three design patterns was carried out in a standard microwave chamber room. The experimental configuration is illustrated in Figure 5. A broadband horn antenna at a certain distance from the metasurface sample was used as the feed source and was fixed onto the workbench together with the metasurface specimen. Another broadband horn antenna was placed 10 m away from the metasurface sample as a receiver. With the rotation of the metasurface specimen on the workbench, the two-dimensional far-field data can be measured.

**Figure 6:**
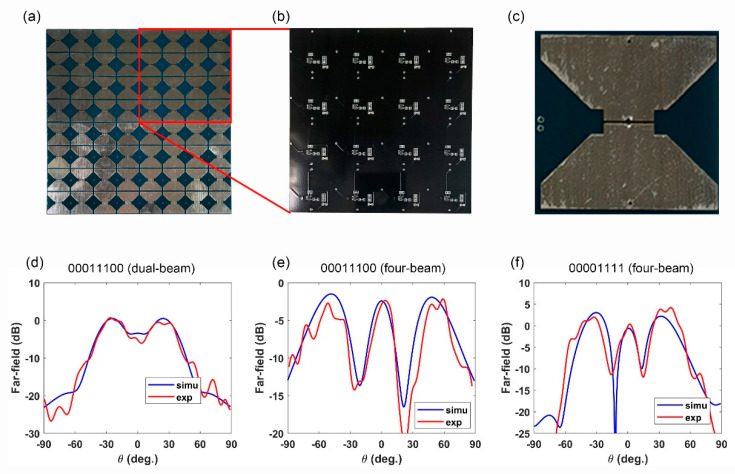
The details of the metasurface sample and the far-filed results. (**a**) A photograph of the sample shows the details of the fabricated metasurface sample, and the components distributed on it; (**b**) the soldering and circuit distribution of the microcircuit; (**c**) the top view of the sample meta-cell; (**d**–**f**) comparison of the simulated and measured far-field results of the metasurfaces for the three coding patterns.

The details of the metasurface sample are shown in Figure 6a. The sample is composed of 8 × 8 elements and is integrated with 64 PIN diodes. The entire metasurface board is made up of 4 identical 4 × 4 submodules, and one submodule is shown here for circuit soldering and distribution. The structure formed by these submodules can be applied to electromagnetic imaging and stealth scenes based on thermal perception and has great prospects for radar detection. Thermistors are embedded in each metasurface element to ensure that each unit cell can be modulated. In order to isolate the RF energy, RF chokes are used, and the bias hole is connected to the voltage drive module through two RF chokes. At the same time, distinct thermal distributions are obtained by using small metal plates with electric heaters inside. The soldering and circuit distribution of the microcircuit are shown in Figure 6b. Figure 6c also shows the top structure of the metacell.

To facilitate the comparison between the simulation results and the experimental results, we used blue and red lines to represent the far-field results of the simulation and experiment of three patterns at 3 GHz, respectively. From Figure 6d–f, we can clearly observe that the line trends of the two colors are roughly the same, which proves that the simulated results are highly consistent with the experimental results. In addition, we can observe the metasurface array coding “00011100” reflects the incident wave into a dual-beam, and the chessboard metasurface horizontal coding “00011100” and “00001111” reflects the incident wave into a quadruple-beam with different reflection angles. There are minor errors in the results, mainly due to the following two aspects: (1) the PIN diode is regarded as an RLC model in the simulation, which is not completely consistent with the equivalent model in the experiment; (2) there are manual errors and manufacturing errors in the experiment; (3) the nonideal excitation of the horn antenna since it is not an ideal plane-wave.

## 3. Conclusions

A programmable metasurface integrated with PIN diodes and thermistors is presented. Each metasurface unit cell is embedded with a thermistor, which can sense the heat distribution independently. We can modulate the electromagnetic wave by controlling the arbitrary metasurface elements and realizing the distinct beam deflection on the metasurface under diverse coding sequences. In order to verify the performance of the metasurface, three different patterns were designed, and their far-field results were simulated and measured under specific thermal distribution. Through comparison, the results of the simulation and experiment are in good agreement. This work enables the metasurface to modulate electromagnetic waves with more flexibility, and the metasurface has the characteristics of low cost and high performance.

## Data Availability

Not applicable.
